# Friedreich's Ataxia, Frataxin, PIP5K1B: Echo of a Distant Fracas

**DOI:** 10.1155/2013/725635

**Published:** 2013-09-30

**Authors:** Aurélien Bayot, Pierre Rustin

**Affiliations:** ^1^INSERM UMR 676, Bâtiment Ecran, Hôpital Robert Debré, 48 boulevard Sérurier, 75019 Paris, France; ^2^Université Paris 7, Faculté de Médecine Denis Diderot, Site Robert Debré, 48 boulevard Sérurier, 75019 Paris, France

## Abstract

“*Frataxin fracas*” were the words used when referring to the frataxin-encoding gene (*FXN*) burst in as a motive to disqualify an alternative candidate gene, *PIP5K1B*, as an actor in Friedreich's ataxia (FRDA) (Campuzano et al., 1996; Cossee et al., 1997; Carvajal et al., 1996). The instrumental role in the disease of large triplet expansions in the first intron of *FXN* has been thereafter fully confirmed, and this no longer suffers any dispute (Koeppen, 2011). On the other hand, a recent study suggests that the consequences of these large expansions in *FXN* are wider than previously thought and that the expression of surrounding genes, including *PIP5K1B*, could be concurrently modulated by these large expansions (Bayot et al., 2013). This recent observation raises a number of important and yet unanswered questions for scientists and clinicians working on FRDA; these questions are the substratum of this paper.

## 1. Friedreich's Ataxia

With an estimated prevalence of 1 : 50,000 and a carrier frequency of about 1 : 60 to 1 : 90, Friedreich's ataxia (FRDA) is the most commonly inherited ataxia in the Caucasian population [1, 2]. It is a multisystemic, degenerative disease typically associated with dysarthria, muscle weakness, spasticity in the lower limbs, scoliosis, bladder dysfunction, absent lower limb reflexes, loss of position and vibration sense, and speech and listening difficulties [3]. A majority of the affected individuals have hypertrophic cardiomyopathy. Glucose intolerance and diabetes mellitus are observed in a subset (about 30%) of cases. The onset of symptoms is usually between 10 and 15 years of age, but either much earlier or later onset has been infrequently observed. Initial symptoms can be purely neurological, but occasionally, cardiomyopathy can be the presenting symptom. Altogether, atypical presentations represent as much as 25% of the cases [4]. 

## 2. The Molecular Mechanism

In more than 98% of the cases, the disease originates from large homozygous GAA repeat expansions (66 to 1700 repeats; normal: 5 to 33, with 85% less than 12) in the first intron of *FXN* encoding a mitochondrial matrix targeted protein, frataxin. In between uninterrupted expansions, 34 to 66 represent premutation, or borderline alleles, at risk for intergenerational expansion. The few residual cases represent compound heterozygous for an expanded allele and a point mutation, most frequently a null mutation [5].

## 3. Frataxin Depletion: Iron-Sulfur Cluster Deficiency

Simultaneously to the discovery of the molecular basis of FRDA by the European consortium combining the teams of Koenig and Pandolfo [6], shutting down debate on the origin of the disease [7, 8], we studied an endomyocardial biopsy of a young girl, undiagnosed at the moment of the investigation, presenting with massive cardiac hypertrophy but hardly detectable neurological signs [9]. We evidenced a specific and severe deficiency of the activity of all the studied enzymes harbouring iron-sulfur cluster (ISC), namely, mitochondrial respiratory chain complexes I, II, and III and both Krebs cycle mitochondrial and cytosolic aconitase. As this little girl was later on shown to be homozygous for expanded FRDA alleles, we investigated a few more FRDA cases and established that expansion in *FXN *actually caused an activity defect of ISC-containing proteins (ISPs) in the hypertrophied heart of patients [9]. The generalized loss of ISP activity thereafter found its explanation, when the role of frataxin in the mitochondrial biogenesis of ISC was demonstrated in the yeast *Saccharomyces cerevisiae *[10] and human cells [11, 12] by the group of R. Lill.

## 4. Low Frataxin, Normal Iron-Sulfur Clusters: What Causes Hypersensitivity to Oxidative Stress?

However, detailed investigation of affected persons showed that ISPs from circulating lymphocytes, lymphoblastoid cell lines, skeletal muscle, and skin fibroblasts were curiously spared despite low frataxin levels. In the absence of ISP deficiency, a hypersensitivity to oxidative stress was nevertheless frequently observed as a result of expansion in the *FXN *gene, associated in some cells with cytoskeleton anomalies [13]. Obviously these later cytosolic anomalies could not be easily reconciled with the putative intramitochondrial location and function of the frataxin in the ISC biogenesis [14], which moreover did not appear significantly altered in these tissues. 

To account for this puzzling observation, we focused our attention on frataxin-deficient FRDA patient fibroblasts that displayed normally functional ISP. Studying these cells, we could first ascribe the hypersensitivity to oxidative stress to impaired signaling of antioxidant defenses [15]. We further ascribed this impairment to a mislocation and a decrease of the NRF2 (nuclear factor erythroid 2-related factor 2) transcription factor [16]. This mislocation was concomitant with an abnormal remodeling, already noticed in these cells [17], of the actin fibers known to bind NRF2 through interaction with Keap1 (Kelch-like ECH-associated protein 1). But this only displaced the question, what could be the link between the depletion of the mitochondrial frataxin protein and the abnormal status of the cytosolic actin network [13]? This was an incentive to reexamine the whole series of potential consequences of the GAA expansion from the very first event, the gene modification itself. 

## 5. GAA Expansions in *FXN*: Which Consequences?

Rather than to focus our effort on the *FXN* gene only, the idea came to extend the study to the adjacent genes, inspired by the increasingly recognized importance of epigenetics in nucleotide repeat expansion disorders [18]. To our surprise, in patients' cells (skin fibroblasts and circulating lymphocytes), the expansion was found not only to impair the transcription of the frataxin gene, but also that of the adjacent *PIP5K1B* [19]. The decrease was somewhat less pronounced than what could be observed for the frataxin gene, with rare (1/5) fibroblast cultures where the decrease was weaker than expected from the size of the expansion. Nevertheless, we could establish that it had a major impact on the cell actin network, and this was observed in the absence of any detectable consequence of the frataxin depletion on ISPs. Extensive cell biology studies established that PIP5K1B depletion impaired phosphatidylinositol 4,5-bisphosphate [PI(4.5)P_2_] synthesis, causing actin fiber disorganization and a number of associated structural anomalies, including significant delay in cell spreading.

These observations raise a number of questions dealing with (1) the mechanism of gene extinction in the disease, (2) the potential consequence(s) of the PIP5K1B depletion in the expression and course of the FRDA disease, and (3) with therapeutic strategies to be adopted.

## 6. The Mechanisms of *PIP5K1B* Decreased Expression

**Figure 1:**
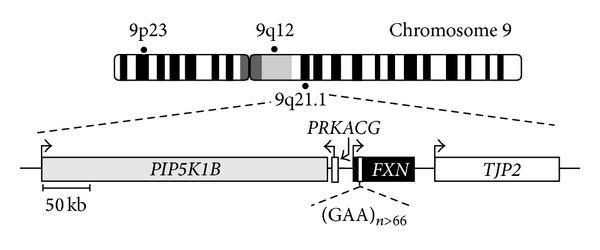
Map of chromosome 9 and the 549.5-kb region containing the human *FXN* and its close neighboring genes. The intronic site of GAA expansions causing FRDA is indicated in *FXN* and gene transcription orientation is shown by black arrows.

We previously established that *PIP5K1B* expression decrease was largely correlated with the size of GAA expansion in *FXN*, and that both PIP5K1B mRNA and protein were concomitantly decreased [19]. To date at least twenty neurological diseases are caused by genomic expansions of repeats in protein-coding or noncoding regions of the genome [20]. The noncoding expansions often result in complex disease phenotypes which may be either developmental and/or degenerative. In FRDA, it was first proposed that the expansion positioned two kilobases from the frataxin promoter in the first intron of the gene tends to form triplex DNA structures which may contribute to inhibiting transcriptional elongation [21]. Later on, relatively short GAA expansions of 200 repeats have been shown to confer variegated gene silencing of a linked mouse transgene marker, similar to position-effect variegation [22]. Then, GAA repeat expansions have been associated with a number of epigenetic changes [23], including (1) di- and trimethylation of H3K9, together with hypoacetylation of H3K14, H4K5, H4K8, and H4K12 and (2) an increase in DNA methylation at specific CpG sites in the region of *FXN* intron 1 immediately upstream of the GAA repeat. The direct effect of GAA expansions on the generation of local transcriptionally inactive chromatin was demonstrated by the use of histone deacetylase (HDAC) inhibitors, molecules known to increase global histone acetylation and thereby reactivate epigenetically silenced genes [24]. In FRDA patients' cells, such compounds led to an increased acetylation of several histone proteins at the *FXN* locus, including H3K14, H4K5, and H4K12 and to a significant reversion of *FXN* silencing [25]. Additional testing of selective HDAC inhibitors in appropriate mouse models further reported positive effects, including a reversion of specific repressive histone marks, a significant correction of frataxin levels, and an improvement of some phenotypic features [26–28]. Parallel to the formation of local heterochromatin, further work has pointed out that some heterochromatin marks propagate into the *FXN* promoter, thus supporting the idea that in addition to impairing elongation of gene transcription at the repeat site [29, 30], GAA expansions may also interfere with transcription initiation at the *FXN* locus [31]. In keeping with this, an intriguing finding is the observation that depletion of CTCF, a highly conserved multifunctional transcription regulator and chromatin organizer, in the 5′-UTR of expanded *FXN* alleles may initiate spreading of repressive chromatin from GAA expansions by increasing the expression of an antisense transcript (FAST-1) [32]. Altogether, these data support the view that a heterochromatinization process at the *FXN* locus, consisting of the formation and local spreading of heterochromatin, compromises optimal access/progress of the transcription machinery and possibly of transcription factors for correct *FXN* gene expression. Both chromatin reorganization and one or more of the abovementioned epigenetic changes might also account for the reduced expression of the adjacent *PIP5K1B* gene. It is too early to decipher a mechanism accounting for *PIP5K1B* decreased expression, and it remains to be shown that the expression of these two genes only is affected, as other genes are present in the vicinity (Figure 1). 

## 7. The Potential Consequence(s) of the *PIP5K1B* Depletion in the FRDA Disease?

The consequences of frataxin depletion have been a matter of discussion since the early days of its discovery. According to authors, frataxin might be or act as mitochondrial iron export carrier [33], a source or a trap for mitochondrial iron acting as a gate keeper for ISC synthesis [34], an instrumental component of the ISC synthesis machinery [10], an ISC chaperone [35], or an antioxidant factor [36]. All of these hypotheses have been indeed supported by more or less convincing experimental data, sometimes hardly reconcilable, recently discussed in details [37]. Adding to these interrogations, the recently described phenotypes of diseases originating from mutations in genes instrumental in ISC synthesis are surprisingly enough, not significantly overlapping with FRDA [13, 38].

**Figure 2:**
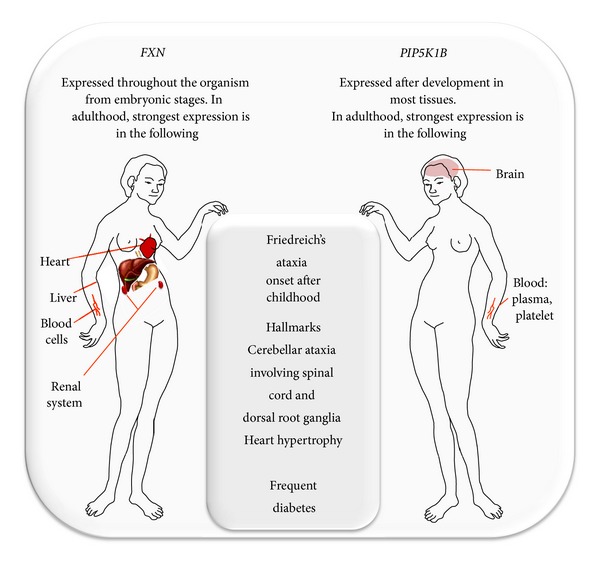
Territories of the strongest expression of *FXN* and *PIP5KB* in human. Data for human healthy tissues from MOPED (Model Organism Protein Expression Database) and PaxDb (Protein Abundance Across Organisms).

Considering that the triplet expansion in *FXN *can also hamper *PIP5K1B *expression, it might as well be that part of the biochemical phenotypes reported in human cells/samples are not caused by the frataxin depletion only. PIP5K1B participates in the biosynthesis of PI(4,5)P_2_ and is expressed, essentially after childhood, in a number of tissues in human, highest expression being reported in the brain (Figure 2). PIP5K1B is at the crossroad of different signaling pathways, mediating RAC1-dependent reorganization of actin filaments and contributing to the activation of phospholipase D2 [39, 40]. Considering the pleiotropic role in the cell of PIP5K1B, it is not easy to predict what would be, presumably dependent on cell type, the consequences of a partial depletion of the protein, not necessarily restricted to actin network remodeling. Moreover, the consequences of PIP5K1B depletion might well be mixed with the consequences of frataxin deficiency. Indeed, when occurring, impairment of ISC synthesis resulting from frataxin depletion predictably has also a number of various consequences. In addition to affecting the activity of the respiratory chain and of the Krebs cycle enzyme, aconitase, this will impair the overall ISC homeostasis in the cell, impacting on a number of cytosolic enzymes containing ISC, and in turn this might ultimately affect organic acid balance, iron homeostasis, and oxidative stress sensitivity. Obviously, each of these factors can have unpredicted effect on cellular metabolism. For example, discrete metabolic disturbance increasing electrophile acids may modify cysteine residues of Keap1 that links NRF2 and actin [41, 42]. The result will be a constitutive activation of NRF2 that could on a long term exhaust the antioxidant capacities of the cell and results in hypersensitivity to oxidative stress. Similar impairment can possibly result from PIP5K1B depletion since this latter has been shown to trigger abnormal remodeling of the actin network known to control NRF2-ARE activation [43]. Either frataxin or PIP5K1B depletion, or both, might thus contribute to the oxidative-stress hypersensitivity observed in FRDA [44].

Timing (after birth) and territories of *PIP5K1B* expression are compatible with the course and presentation of FRDA (Figure 2). Predicted cellular consequences of PIP5K1B depletion, especially actin network remodeling, are sufficiently severe as to be instrumental in the pathology. However it would be premature to speculate on the potential role of PIP5K1B depletion in the disease, as depletion is yet to be demonstrated in affected tissues (especially brain and heart). 

## 8. Impact on Present and Future Therapeutic Strategies

**Figure 3:**
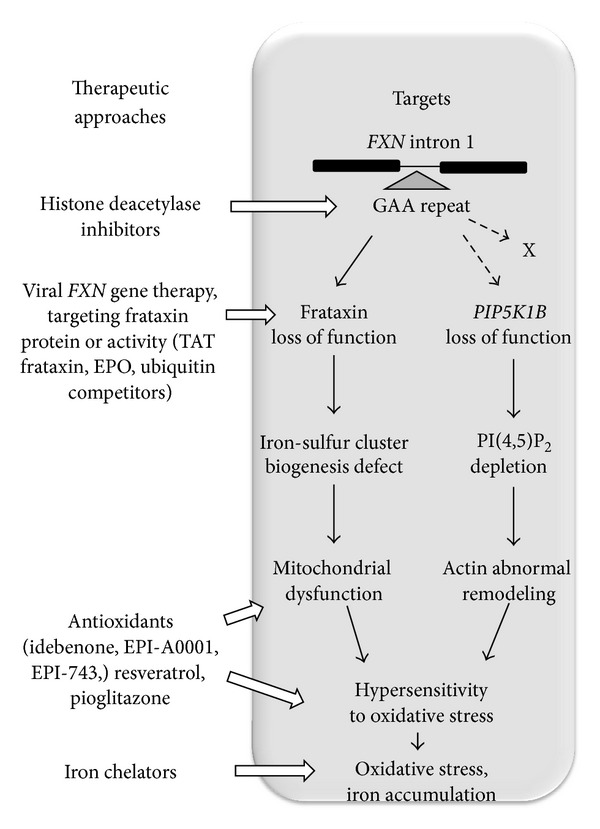
The therapeutic strategies envisioned to fight Friedreich's ataxia and their targets.

Strategies targeting different steps of the disease have been proposed/tested to counteract FRDA (Figure 3). A very promising approach is to target the GAA expansion using effective reagents for deacetylation of histones “wrapping” the expansion as to facilitate gene reading [24]. Obviously, the hypothesis that the expansion hampers more than frataxin expression does not modify the rationale of this approach. 

Then a series of approaches aim at restoring the level of frataxin, either by bringing the gene or by targeting posttranscriptional, or even post translational steps, in both frataxin synthesis or activity [45]. Depending on the actual impact of frataxin depletion versus that of PIP5K1B, the importance to restore frataxin level/activity might be more or less crucial. However for the time being, we have all reasons to believe that, even if variable according to tissues, and possibly time, the impact of frataxin depletion is instrumental in the disease, for example, severe ISC deficiency evidenced in the heart of affected persons. Restoring frataxin level thus remains a major issue, even if other actors might be shown to play a role in the disease. For example, the hypersensitivity to oxidative insult might result from the concurrent depletion of frataxin and PIP5K1B, and increasing frataxin level might as well be sufficient to restore the proper capacity of antioxidant defenses. 

Last, strategies targeting one of the supposedly major downstream effects of frataxin depletion, that is, hypersensitivity to oxidative insult, have been proposed [46–48] and, for some of them, tested since nearly 15 years [49]. So far, clinical trials (mostly with idebenone) have resulted in unsatisfactory, partial, and contradictory results. We are left with the conclusion that was reached in our early study: the antioxidant treatment, having no spectacular effect on the ataxia, decreases the heart mass, improving fatigability, thin movements, and speech fluency, although in a subset of the affected persons only [50, 51]. As discussed above, the hypersensitivity to oxidative stress may possibly stand from the PIP5K1B depletion as well, not weakening in any sense the rational to use antioxidants in FRDA. 

Interestingly enough a number of strategies have been devised based on experiments attempting to modulate the phenotype (sensitivity to oxidative stress) of FRDA patients' fibroblasts [52–54]. We now know that the phenotype of these cells, variably severe from individual to individual, does not result from a putative ISC depletion induced by decreased frataxin, but is most probably caused by PIP5K1B depletion. Obviously, the question of the model to be used to screen for therapeutic strategies will become a central one if PIP5K1B depletion is shown instrumental in the disease. By definition, the numerous organisms—flies, worms, and mice—or cells—HeLa cells, induced pluripotent stem cells, etc.—that have been engineered by specifically targeting frataxin, cannot pretend to represent the complexity that can be envisioned if more than frataxin is affected in the disease. 

## 9. Concluding Remarks

The identification of PIP5K1B as a potential actor in the pathological process underlying FRDA obviously raises a number of questions that urgently need to be answered. The very first of these is the actual implication of the PIP5K1B depletion in the disease onset and course. To date, decreased PIP5K1B has been shown in circulating blood cells and cultured skin fibroblasts in FRDA [19]. To further consider an implication in the disease, a decreased expression of the gene should be established in at least one affected tissue. The mechanism of decreased *PIP5K1B *expression is a second important question to answer, especially as depending on the mechanism involved, expression of additional genes in the region might be modulated as well. It is noticeable that because we are dealing with subtle epigenetic modulations, the phenomenon might be affected by a number of interfering factors possibly variable among individuals and conditions. In any case, these epigenetic modulations might possibly account in part for the poor correlation between size of the expansions and severity of the disease. Many other factors may explain the variability observed in the disease. To mention only one, in FRDA as in most degenerative diseases which are primarily systemic bioenergetics diseases, mitochondria and their confined genetic material, prone to mutations, presumably participate in the clinical heterogeneity [55].

Our last concluding remark deals with the consequence in term of therapeutic strategies. As detailed above, none of the actual therapeutic approaches should be invalidated. Indeed, if PIP5K1B depletion proves to be instrumental, then it might provide additional targets to fight FRDA and allow devising new therapeutic strategies.
